# Cost-Efficient and Easy to Perform PCR-Based Assay to Identify Met Exon 14 Skipping in Formalin-Fixed Paraffin-Embedded (FFPE) Non-Small Cell Lung Cancer (NSCLC) Samples

**DOI:** 10.3390/diagnostics9010013

**Published:** 2019-01-18

**Authors:** Odharnaith O’Brien, Mark C. Wright, Cathal O’Brien, Orla Geoghegan, Niamh Leonard, Siobhan Nicholson, Sinéad Cuffe, Aurelie Fabre, Wolfram Jochum, Markus Joerger, Steven G. Gray, Stephen P. Finn

**Affiliations:** 1 Thoracic Oncology Research Group, Trinity Translational Medicine Institute, St. James’s Hospital, D08 W9RT Dublin, Ireland; odharnaithobrien@gmail.com (O.O.); geoghego@tcd.ie (O.G.); scuffe@stjames.ie (S.C.); sgray@stjames.ie (S.G.G.); 2 Department of Histopathology, Labmed Directorate, St. James’s Hospital, D08 RX0X Dublin, Ireland; mwright@stjames.ie (M.C.W.); nleonard@stjames.ie (N.L.); snicholson@stjames.ie (S.N.); 3 Cancer Molecular Diagnostics, Labmed Directorate, St. James’s Hospital, D08 RX0X Dublin, Ireland; OBRIEC12@tcd.ie; 4 HOPE Directorate, St. James’s Hospital, D08 RT2X Dublin, Ireland; 5 Department of Pathology, St. Vincent’s University Hospital, University College Dublin School of Medicine, D04 T6F4 Dublin, Ireland; afabre@svuh.ie; 6 Department of Pathology, Cantonal Hospital, 9007 St. Gallen, Switzerland; wolfram.jochum@kssg.ch; 7 Department of Medical Oncology & Hematology, Cantonal Hospital, 9007 St. Gallen, Switzerland; Markus.Joerger@kssg.ch; 8 Department of Clinical Medicine, Trinity College Dublin, D02 PN40 Dublin, Ireland; 9 School of Biological Sciences, Dublin Institute of Technology, D08 NF82 Dublin, Ireland; 10 Department of Histopathology and Morbid Anatomy, Trinity College Dublin, D08 X4RX Dublin, Ireland

**Keywords:** Met exon 14 skipping, diagnostic assay, PCR, next generation sequencing, RNA hybridisation

## Abstract

MET is a receptor tyrosine kinase (RTK) that plays important roles in carcinogenesis. Despite being frequently overexpressed in cancer, clinical responses to targeting this receptor have been limited. Recently novel splicing mutations involving the loss of exon 14 (called METex14 skipping) have emerged as potential biomarkers to predict for responsiveness to targeted therapies with Met inhibitors in non-small cell lung cancer (NSCLC). Currently, the diverse genomic alterations responsible for METex14 skipping pose a challenge for routine clinical diagnostic testing. In this report, we examine three different methodologies to detect METex14 and assess their potential utility for use as a diagnostic assay for both the identification of METex14 and intra-tumoural distribution in NSCLC.

## 1. Introduction

Non-small cell lung cancer (NSCLC) is a heterogeneous histological and molecular entity. The discovery of actionable mutations present within a subset of these tumours, most notably mutations in Epidermal Growth Factor Receptor (*EGFR*), and rearrangements of anaplastic lymphoma kinase (*ALK*) and V-Ros avian UR2 sarcoma virus oncogene homolog 1 (*ROS1*), has led to the development of a number of effective targeted treatment options for patients with advanced disease [1].

MET protooncogene (*MET*) also known as Hepatocyte Growth Factor Receptor (*HGFR*) codes for a receptor tyrosine kinase (RTK) that plays a role in tissue remodelling and morphogenesis. Its only known ligand is hepatocyte growth factor/scatter factor (HGF/SF), whose binding results in physiological activation of MET [2]. Its activation results in downstream induction of the Phosphatidylinositol 3-kinase/mechanistic target of rapamycin (PIK3/mTOR), signal transducers and activators of a transcription (STAT) and Mitogen-activated protein kinase (MAPK) pathways. Abnormal activation of MET results in increased cell survival, cell proliferation, epithelial–mesenchymal transition (EMT), invasion and angioinvasion and ultimately, the promotion of oncogenesis. Alterations of MET, such as protein overexpression, gene amplification and mutations in MET gene juxtamembrane and semaphorin domains have been observed in a variety of cancers and are associated with a poor prognosis in NSCLC. A splicing mutation involving the loss of exon 14 (called METex14) is a recently described biomarker that has emerged as a further potential therapeutic target in patients with NSCLC. The presence of METex14 skipping has been shown to predict whether or not patients may respond to MET inhibitor therapy [3]. In this regard, patients with METex14 skipping have been found to be sensitive to MET inhibitors such as crizotinib, whereas MET protein overexpression or gene amplification results in no durable response to targeted therapy. This may go some way to explaining why trials with MET inhibitors have proved disappointing [4,5]. The sensitivity of METex14 skipped patients to MET inhibitors has resulted in several case reports of dramatic responses in the literature [6,7], and various clinical trials of MET tyrosine kinase inhibitors in METex14 mutated NSCLC are consequently currently ongoing [4]. METex14 skipping is also associated with varying clinical phenotypes depending on the histologic subtype of the tumour encountered. For example, Kwon et al., looking at cohorts of patients with MET-mutated adenocarcinoma (9/102) and pleomorphic carcinoma (9/45), found that patients with MET-mutated adenocarcinomas were significantly older than those without MET mutations (*p* = 0.015) and were twice as likely to be female (male to female ratio: 3:6) and to have never smoked (ever-to never-smoker ratio: 2:7) [8]. All MET-mutated adenocarcinomas in the study (9/9) showed acinar predominant histology with associated lepidic patterns. In contrast, among patients with MET-mutated pleomorphic carcinomas, the male to female and ever- to never-smoker ratios were 8:1 and 7:1, respectively [8].

METex14 alterations have also been identified in 3–4% of non-small cell lung cancers (NSCLC) [9], most frequently in lung adenocarcinomas but also in squamous cell carcinomas. Specific mutations resulting in METex14 skipping are responsible for in-frame deletion of the MET juxtamembrane domain of the MET receptor, which contains the CBL E3-ubiquitin ligase-binding site. This leads to inhibition of degradation of the MET receptor, prolonging its activity [10]. Interestingly, a high incidence of METex14 skipping has been reported in sarcomatoid carcinoma of the lung, although the incidence varies between studies (ranging from 3% up to 31.8% of cases) [11,12,13,14,15,16,17,18,19]. Sarcomatoid lung carcinoma is a rare form of lung carcinoma, accounting for approximately 0.3–1.3% of all lung malignancies. It comprises a group of poorly differentiated NSCLCs that exhibit areas of sarcoma or sarcoma-like differentiation, and encompasses 5 histologic subgroups—pleomorphic carcinomas, spindle cell carcinomas, giant cell carcinomas, carcinosarcomas and pulmonary blastomas. Clinically, it is associated with a poor prognosis and a reduced response to chemotherapeutic agents [20].

From a diagnostic pathology perspective, the identification, where technically possible, of treatment predictive biomarkers in situ within tumour cells must be highly reproducible, particularly when using well validated immunohistochemistry [21,22]. Immunohistochemistry has unfortunately not proven useful thus far for the detection of METex14 splice mutations. MET antibodies are not specific for the METex14 splice variant mutation; they detect MET overexpression, for which there can be numerous causes, e.g., increased gene copy number, gene amplification, METex14 skipping, etc. In addition, a high degree of inter-observer variability has been reported in the scoring of immunohistochemistry (IHC) slides [5]. Recently, the reproducibility and reliability of RNA in situ hybridisation (RISH) has significantly advanced with the advent of novel in situ hybridisation techniques, such as the BaseScope^TM^ methodology developed by ACDbio. This technology has been subsequently optimized for the detection of MET exon 14 skipping [23].

RT-PCR is also a validated and effective method for detecting this particular mutation; however, it lacks the in situ visualization component of IHC or RISH [4,10].

In this study we aimed to optimize, validate and subsequently compare a variety of laboratory techniques to reliably detect the presence of METex14 skipping in NSCLC in formalin-fixed paraffin-embedded (FFPE) tissue.

## 2. Materials and Methods

### 2.1. Patient Cohort

Our initial cohort for analysis comprised patients from multiple cancer centres and institutes with NSCLC (total *n* = 6, comprising *n* = 1 (St. James’s Hospital—SJH), *n* = 2 (St. Vincents University Hospital—SVUH) and *n* = 3 St Gallen; Table 1, Cohort 1) whose tumours had been confirmed to possess METex14 skipping mutations by Next Generation Sequencing (NGS), currently the gold standard method of detection and for which sufficient tissue remained to assess METex14 skipping with additional techniques. As METex14 skipping mutations are relatively rare, we elected to enrich our cohort with patients who had been diagnosed with primary sarcomatoid carcinoma of the lung. A retrospective search of our laboratory’s pathology database identified a further 20 patients diagnosed with primary pulmonary sarcomatoid carcinoma who had undergone surgical resection of their tumour from 2011 to 2017 (Table 1, Cohort 2). FFPE tissue from these patients’ tumours was used to detect the presence or absence of METex14 skipping.

### 2.2. Ethics

All patients gave their informed consent for inclusion before they participated in the study. This study was conducted in accordance with the Declaration of Helsinki. Studies were completed on FFPE specimens with approval from the relevant Hospital Ethics Committees (SJH/AMNCH—# 041018/8804—approval date 24/10/2004, SVUH Protocol, PIL/Consent vs 4—approval date 21/04/2009 and St Gallen EKSG 17/013).

### 2.3. Polymerase Chain Reaction (PCR)

A one-step RT-PCR end-point PCR assay to examine for the detection of METex14 skipped mRNAs in FFPE was designed, optimized and tested in this enriched cohort of NSCLC patients. Initial attempts at performing PCR found that standard end-point PCR resulted in a significant rate of false positive results. However, this issue was resolved with the introduction of one-step RT-PCR methodology using a Verso 1-Step RT-PCR kit (Thermo Fisher Scientific, Waltham, MA, USA; Cat No. AB-1454/LD/A). This kit incorporates a Taq DNA polymerase that has reverse transcriptase activity with high processivity at temperatures of up to 57 °C, delivering effective reverse transcription through difficult RNA secondary structures. Total RNA was isolated from freshly cut 5 μm thick FFPE sections, or directly from slides using a Qiagen RNeasy ©FFPE kit (Qiagen, Germantown, MD, USA; Cat No./ID: 73504), and this was quantified with a nanodrop-1000 (Thermo Fisher Scientific, Waltham, MA, USA). One hundred nanograms was subsequently examined for the presence of Met exon14 skipping using primers spanning exons 13–15 by means of a Verso 1-Step RT-PCR kit (Thermo Fisher Scientific, Waltham, MA 02451, USA Cat No. AB-1454/LD/A). To assess for the presence of METex14 skipped transcripts, 100 ng of total RNA was subjected to 1-step RT-PCR using the following primers:

METex14 FWD: 5′-TTGGGTTTTTCCTGTGGCTG-3′

METex14 REV: 5′-GGATACTGCACTTGTCGGCA-3′.

To assess for the presence of 18S rRNA, 100 ng of total RNA was subjected to 1-step RT-PCR using the following primers:

18S rRNA FWD: 5′-GATGGGCGGCGGAAAATAG-3′

18S rRNA REV: 5′-GCGTGGATTCTGCATAATGGT-3′.

The PCR cycling conditions for 1-step RT-PCR were as follows: cDNA synthesis at 50 °C for 15 min, followed by Verso Enzyme inactivation at 95 °C for 2 min; then, 45 cycles of amplification with denaturation at 95 °C for 20 s, annealing at 60 °C for 30 s, extension at 72 °C for 60 s and a final extension step at 72 °C for 5 min.

All PCR products were electrophoresed on 2% agarose gel. The PCR products expected using these primers were as follows; Met WT: 235 bases; METex14: 94 bases; and 18S rRNA: 165 bases. For each MET specific PCR, a positive wild-type (WT) control (A549) and a positive METex14 control (NCI-H596) were included, along with negative controls.

The sensitivity of the RT-PCR assay was assessed using GBlocks engineered to exactly mimic the wildtype and Met exon 14 sequences. For the GBlock sensitivity assay, GBlocks were synthesized by IDTdna (Integrated DNA Technologies, Inc. Skokie, Illinois 60076, USA), reconstituted to a final concentration of 10 ng/µL and mixed in the following proportions according to Table 2 to create the following standard curve.

The PCR protocol was modified to remove the cDNA synthesis step, and the assay was run using 5 ng of diluted GBlock. When diluted with decreasing amounts of METex14, the sensitivity of the assay using the Verso 1-Step kit is approximately 10%. A similar assay was subsequently conducted using the same strategy by admixing WT and MetEx14 mutated patient RNA in the same percentages, and conducting the assay including the cDNA synthesis step. The only modification was that the final total of RNA in each sample was 100 ng.

### 2.4. Next-Generation Sequencing (NGS)

Cases of sarcomatoid carcinoma which were proven to possess the METex14 splice variant by RT-PCR were subsequently confirmed to be positive by targeted sequencing of these samples by NGS. Library preparation was performed according to the Oncomine Focus assay protocol (Thermo Fisher Scientific, Waltham, MA 02451, USA), starting with 10 ng of DNase-treated RNA. Samples were multiplexed using IonCode barcode adapters (Thermo Fisher Scientific, Waltham, MA, USA). Final RNA libraries were quantified by quantitative PCR by using the Ion Library Quantitation Kit (Thermo Fisher Scientific, Waltham, MA, USA) and then diluted to 100 pM and pooled. Templating reactions and chip loading were performed using the Ion 510™ & Ion 520™ & Ion 530™ Chef Kit (Thermo Fisher Scientific, Waltham, MA, USA) and sequenced using 200 bp sequencing with Ion 530™ chips. Sample de-multiplexing and initial quality controls were performed on the Torrent Suite Server version 5.4 (Thermo Fisher Scientific, Waltham, MA, USA). Demultiplexed data were uploaded to a local Ion Reporter server for combined DNA and RNA analysis with the Oncomine Focus v2.0 pipeline on Ion Reporter version 5.0 software both Thermo Fisher Scientific, Waltham, MA, USA).

### 2.5. RNA In Situ Hybridisation (RISH)

Using a METex14 skipped cell line (NCI-H596), RISH was optimised and performed on full-face sections of surgically resected tumour from PCR/NGS positive cases using a specific BaseScope™ Assay (Techne/ACDbio, Abingdon, UK). This assay utilises 3 probes in addition to positive and negative controls tested in parallel. One probe, designed to detect the junction between exons 12 and 13, is considered common and can detect both wild-type MET and METex14 skipped mRNA (Techne/ACDbio, Abingdon, OX14 3NB, United Kingdon Cat No. 701821). The second probe is designed to detect the junction between exons 14 and 15 (Techne/ACDbio, Abingdon, OX14 3NB, United Kingdon Cat No. 701811) and detects only MET mRNA containing exon 14 (wild-type MET). The third probe is designed to detect the junction between exons 13 and 15 (Techne/ACDbio, Abingdon, OX14 3NB, United Kingdon Cat No. 701801) and detects only METex14 skipped mRNA. Briefly, 3 µm sections of FFPE tumour were deparaffinised by submersion in xylene and washed with 100% ethanol. Target antigen retrieval was performed by incubating the deparaffinised sections at 100 °C for 15 min. Sections were then rinsed in dH_2_O, dipped in 100% ethanol for 3 min and air dried. A hydrophobic barrier was applied surrounding the tissue on the slide. Suitable positive and negative controls, in addition to the relevant probes described above, were added to the deparaffinised tissue sections and successive amplification steps were performed. A fast red dye was applied to the tissue sections for signal detection. The slides were subsequently analysed and scored by light microscopy.

## 3. Results

### 3.1. Reverse Transcription Polymerase Chain Reaction (RT-PCR)

**Figure 1:**
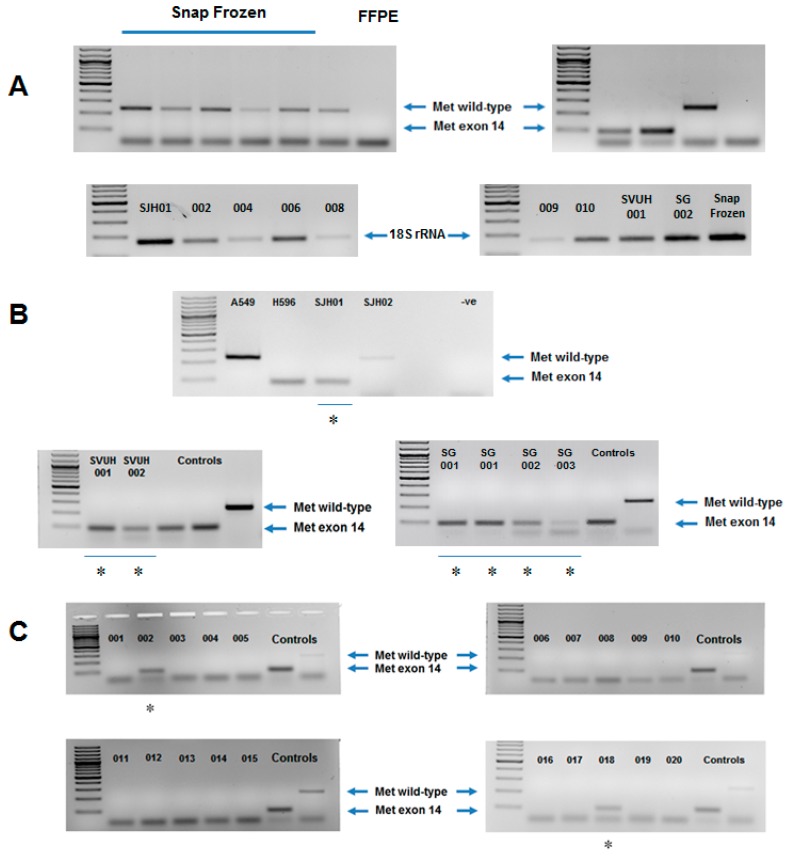
End-point PCR detection of METex14 in three cohorts of non-small cell lung cancer (NSCLC) formalin-fixed paraffin-embedded (FFPE). (**A**) Amplification of the Wild-Type amplicon is limited primarily to high-quality RNA. Samples from snap-frozen tumour tissues versus a sample isolated from an FFPE embedded specimen show that the integrity/quality of RNA is important for detecting WT MET in FFPE embedded samples. A subset of FFPE samples were subsequently examined for the expression of 18S rRNA. Amplification was observed across all specimens; (**B**) Confirmation of the specificity of the MetEx14 assay on FFPE extracted RNA from known METex14 skipped NSCLC cases from three hospitals: St James’s Hospital (SJH); St Vincent’s University Hospital (SVUH) and St Gallen (SG); (**C**) Assessment of a cohort of 20 pulmonary sarcomatoid carcinomas (PSCs) for METex14 skipped samples. * indicates that a given sample exhibits the MetEx14 skipping mutation.

During the optimization of the RT-PCR assay, it became clear that RNA fragmentation during FFPE fixation detection of wildtype MET was, for the most part, undetectable, whilst METex14 was readily detectable due to its smaller amplicon size (Figure 1A). As such, those samples that were negative for any amplification product were deemed wildtype MET. However, to ensure that the lack of amplification was not a consequence of poor RNA quality, we examined a subset of samples for the expression of 18S rRNA. Amplification was observed in all samples (Figure 1A). Once the RT-PCR conditions had been optimized, we then tested our assay on a “training cohort” comprising samples (*n* = 6) known to be METex14 skipped, and confirmed the specificity of this assay on FFPE extracted RNA from known METex14 skipped NSCLC cases isolated from three different centres (Figure 1B).We then tested a “validation cohort” of pulmonary sarcomatoid carcinomas (PSCs) (*n* = 20) to test for the presence of METex14 skipping. We identified METex14 skipped mutations in 10% (2/20) of sarcomatoid patients. The remaining 18 cases (18/20, 90%) were METex14 wildtype (Figure 1C). These results are in agreement with other studies that investigated the incidence of METex14 skipping in sarcomatoid carcinoma [15,16,17,18].

**Figure 2:**
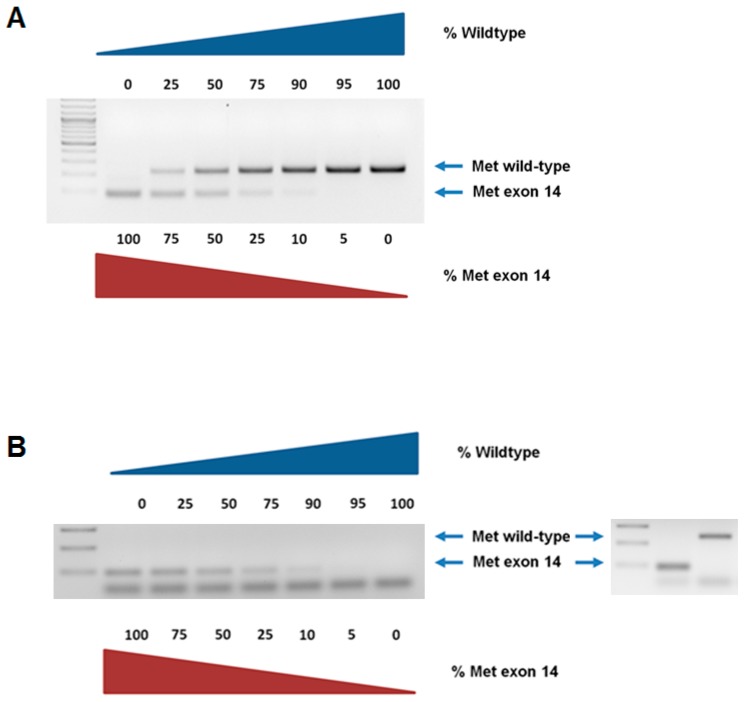
Assay sensitivity for end-point PCR detection of METex14 skipping. Sensitivity to detect MetEx14 was measured using either (**A**) GBlocks or (**B**) admixtures of patient FFPE RNA with limiting amounts/percentages of WT/METex14 as indicated. The limit of detection was found to be 10%.

We then tested the potential sensitivity of this assay using a dilution of WT/METex14 using, in the first instance, synthesized DNA fragments (Figure 2A). Our results determined that the sensitivity of this endpoint RT-PCR could potentially detect METex14 skipped RNA with a lower limit of detection of approximately 10% (Figure 2A). We then tested the potential sensitivity in patient material using the same strategy of dilution for patient admixed RNA, and the results showed a similar sensitivity of 10% (Figure 2B). This therefore indicates that the PCR methodology presented is robust enough to allow for analysis of samples from multiple centres.

### 3.2. Next-Generation Sequencing (NGS)

Cases that demonstrated METex14 skipping by RT-PCR were confirmed using targeted RNA sequencing by NGS. All cases demonstrating METex14 skipping by PCR had a corresponding METex14 skipping mutation identified by RNA sequencing by NGS.

### 3.3. RNA In Situ Hybridisation (RISH)

**Figure 3:**
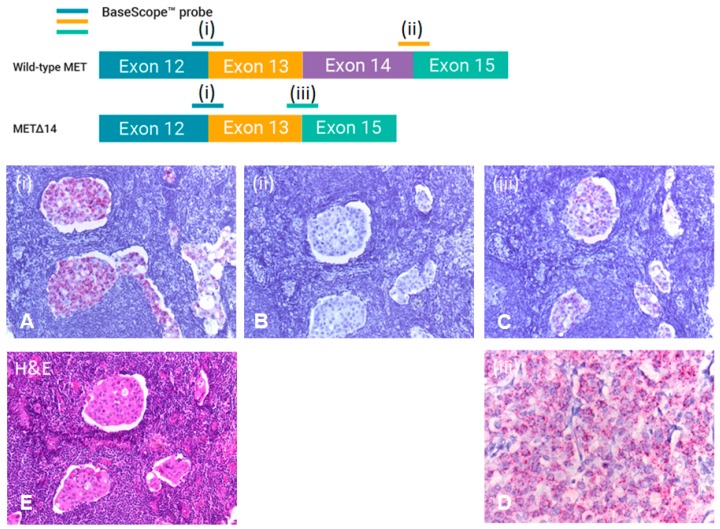
RISH analysis. Probe design for the detection of METex14 using the ACDbio BaseScope™ assay. One probe (blue—(i)), designed to detect the junction between exons 12 and 13, is considered common and can detect both wild-type MET and METex14 mRNA. A second probe (orange) is designed to detect the junction between exons 14 and 15 (ii) and detects only MET mRNA containing exon 14 (wild-type MET). A third probe is designed to detect the junction between exons 13 and 15 (iii) and detects only METex14 skipped mRNA (METex14). The three probes are tested in parallel. An example of RISH on a patient sample using these probes demonstrating the presence of METex14 skipping. 20× magnification of (Panel **A**) exon 12/13 probe (detects all MET); (Panel **B**) exon 14/15 probe (detects wild-type MET); (Panel **C**) exon 13/15 probe (detects (METex14)). In this example, almost the entire sample demonstrates METex14. (Panel **D**) 40× magnification of the (iii) exon 13/15 probe (METex14), while (Panel **E**) is a H&E stain from the same sample.

RISH was performed on FFPE sections taken from resection specimens of patients that were confirmed to harbour a METex14 skipping mutation by RT-PCR and next-generation sequencing. All tested cases were confirmed as METex14 skipped by RISH, with positive signals detected with the probes E13/15 and E12/13, and absence of a detectable signal with the E14/15 probe (Figure 3).

A summary of the patient samples and the results for each assay is presented in Table 1.

## 4. Discussion

The treatment of advanced stage NSCLC has been transformed in recent years by the identification of biomarkers, such as EGFR sensitizing and resistance mutations, ALK and ROS1 rearrangements, PD-L1 expression and tumour mutational burden assessment, allowing clinicians to tailor therapy using tyrosine kinase inhibitors and immunotherapeutic and chemotherapy agents [1,5,24,25]. Most recently, the discovery that certain METex14 mutations are potential drivers of disease in NSCLC, exhibiting sensitivity to treatment with MET-inhibitor therapy, has led to renewed interest in MET as a targetable biomarker. Accurate, reliable and timely detection of these mutations is paramount in patients with advanced NSCLC. Meaningful improvements in progression-free survival have been documented in patients harbouring EGFR mutations and ALK rearrangements [26,27,28] who have received tyrosine kinase inhibitor therapy, and case reports and case series have begun to show this may also be achievable in those patients with METex14 skipping [3,6,7,19,29,30,31,32,33].

Initial phase III clinical trials investigating the effect of MET inhibitors in patients with NSCLC have demonstrated disappointing results [34,35,36]. Significantly, however, these trials targeted patients exhibiting MET overexpression/amplification and did not examine patients with METex14 mutations exclusively. However, as mentioned above, several studies have emerged showing clinical responses in patients with METex14 skipped NSCLC who were treated with MET inhibitors [3,6,7,19,29,30,31,32]. Paik and colleagues prospectively identified eight patients with stage IV lung adenocarcinoma who harboured METex14 splice site alterations [29]. In this study, four patients were treated off-label with MET-inhibitor therapy (three patients received crizotinib, one patient received cabozantinib). All four patients experienced either a partial (*n* = 3) or complete response (*n* = 1) of their tumours (as defined as per Response evaluation criteria in solid tumors (RECIST) criteria) to MET inhibitor therapy [29]. A further study by Frampton et al. [3] demonstrated significant clinical responses in one patient treated with crizotinib (>60% partial response) and in two further patients who were treated with capmatinib as part of a phase II clinical trial (53% and 61% partial responses, respectively) [3]. After demonstrating a high rate of occurrence of the METex14 skipping mutation in a cohort of patients with pulmonary sarcomatoid carcinoma (PSC), Liu et al., described one patient with advanced chemotherapy-refractory PSC who had a dramatic response to treatment with crizotinib [19]. Heist et al., described a patient with metastatic squamous cell carcinoma with confirmed METex14 skipping who was treated with crizotinib. A dramatic treatment response was seen radiologically at 4 weeks, and the response was maintained for at least 6 months [37]. A number of other case reports have documented similar responses in patients with advanced stage NSCLC [6,7,30,31,32].

Robust data on progression-free and overall survival in patients with METex14 skipping treated with MET inhibitor therapy are however lacking. Randomized controlled trials have yet to be performed to specifically address this question. However, combining the results of Paik et al. and Frampton et al., the reported duration of therapy before progression ranged from approximately 3 months to 13 months (*n* = 7 patients) [3,29].

METex14 skipping occurs as a result of exon 14 splice site alterations which demonstrate diverse genomic sequence compositions. The various alterations comprise base substitutions and indels (insertion or deletion of bases in the sequence) in exon 14 slice acceptor sites, splice donor sites and in the −25 bp intronic noncoding region adjacent to the splice acceptor site [3]. Various techniques have been used successfully to detect the presence of METex14 skipping, including RT-PCR and next-generation sequencing, as summarized in Table 3.

High MET expression by immunohistochemistry is correlated with METex14 skipping. However, immunohistochemistry detects MET protein overexpression regardless of the underlying MET abnormality and does not specifically identify METex14 skipped tumours. The European Thoracic Oncology Platform (ETOP) found that in a well-defined NSCLC Lungscape cohort of 1572 patients, both MET amplification and MET gene copy number (GCN) were significantly associated with IHC MET positivity (*p* < 0.001) [5]. Importantly, they found neither MET expression nor GCN status, including amplification and polysomy, were significantly associated with tumour stage and had no impact on the measured outcome. Another issue observed was high inter-laboratory variability in MET IHC analysis. The IHC positivity rate varied between 1.6% and 41.6%, and two centres were found to be significantly over-rating and under-rating respectively [5]. In a further study by Lee et al., that evaluated 17 patients with confirmed METex14 skipping, MET immunohistochemistry had a sensitivity of 100% but a specificity of only 70.4% for detection of the METex14 skipping mutation [38].

RT-PCR is one of the most sensitive techniques currently available for mRNA detection and quantitation. PCR is an effective technique for METex14 detection, as it can specifically target the E13/E15 splice region. It is a reliable, efficient and cost-effective laboratory method and can be performed in a standard histopathology laboratory without the need for specialised equipment. Some limitations to the use of PCR exist—for instance it has been shown previously to be a less sensitive and specific technique for the detection of gene fusion products, such as with *ALK* gene rearrangement, where the large diversity of break-points in EML4/ALK fusion transcripts makes PCR challenging, and, in this situation, the use of fluorescent in situ hybridization (FISH) FISH has proven a superior testing method [24]. However, the METex14 splice variant has been successfully detected by quantitative reverse transcription PCR in a number of studies [8,17,19]. Another limitation is that this is an RNA-based assay, and its success relies on the RNA quality in the specimen tested. Inadequate fixation of tissue or prolonged ischaemia of tissue has the potential to induce a false negative result. However, the methodology optimized in this study included the analysis of METex14 on RNA isolated from patient formalin-fixed paraffin-embedded (FFPE) specimens suggesting that this may be a cost-effective and effective rapid screen to test for METex14 skipped patients prior to confirmation using more expensive assays such as targeted NGS or RISH. Such a strategy was recently employed by Kim et al., [9], who used real-time quantitative reverse transcription polymerase chain reaction (qRT-PCR) and Sanger sequencing for their first assessment, followed by next-generation sequencing (NGS; hybrid-capture targeted DNA/RNA sequencing) [9]. One issue in the development of an RT-PCR based assay is the quality of the RNA isolated. Our assay currently assumes that if amplification of MET is negative, then this represents a MET WT sample, but could also indicate that the RNA integrity may be too poor. As can be seen from Figure 1A, we attempted to test this by examining amplification of a different target—in this case, 18S rRNA. Amplification of 18S rRNA was detected in all samples tested, but was non-uniform, suggesting that RNA degradation, as a consequence of the FFPE process, affects the ability of this type of assay to detect larger PCR products in FFPE specimens. It may therefore be essential to design primers that (a) have a smaller amplicon size, and (b) for the purposes of examining WT MET, a second set of primers designed to amplify from exons 13 and 14, or 14 and 15 and having a similar amplicon size to those for METex14 may prove useful for screening and ruling out issues of RNA integrity.

NGS is now a well-developed technology with the capability of identifying METex14 skipping [3,15,16,17,19,39]. A major strength of next-generation sequencing is that it can detect a wide array of genetic abnormalities including substitutions, deletions, insertions, duplications, copy number changes, chromosome inversions and chromosome translocations. It can therefore accurately detect the METex14 splice variant with good sensitivity and specificity. Another advantage is that less DNA or RNA is required to detect these abnormalities than that required for traditional DNA/RNA sequencing methods.

Despite the continuous downward trend in the cost of NGS, the expense of this technology can prove prohibitive to its widespread availability in laboratories. Added to this is the expense of necessary ancillary resources including complex bioinformatics systems, data processing/analysing and data storage capabilities. Using targeted sequencing of genes rather than whole genome sequencing can go some of the way towards reducing the cost of testing [40,41]. In addition, NGS does not afford direct visualisation of the tested tissue, unlike IHC or RNA in situ hybridisation. Therefore, the presence or absence of intratumoral heterogeneity, as recently demonstrated in NSCLC [8,39], cannot be easily determined using NGS, but when there is sufficient coverage, NGS can, to some extent, identify or suggest the presence of intra tumoral heterogeneity by evaluation of the variant allele frequency (VAF).

More recently, RISH has proven to be effective in detecting the METex14 skipping mutation [23]. The BaseScope^®^ VS Reagent Kit (Red) assay used in our study is an RNA in situ hybridisation assay based on patented signal amplification and background suppression technology. The assay uses a proprietary method of in situ hybridisation (ISH) to visualize single RNA molecules per cell in formalin-fixed paraffin-embedded tissue on mounted slides. This assay does not require the RNA-free environment that is required for traditional ISH. The advantage of this technology is that it uses a fast red dye which offers a high contrast between positive signals and the background tissue, making analysis of slides by light microscopy more straightforward and efficient. This is of added benefit where a low copy target gene expression is anticipated (1–20 copies per cell), where the red dot signals are clearly apparent against background hematoxylin staining. Given that recent studies have shown the intratumoral heterogeneity of the METex14 mutation [8,39], RISH represents a suitable technology for the detection of intratumoral heterogeneity within tissue samples.

## 5. Conclusions

Our results demonstrate the optimization of a PCR methodology to robustly detect METex14 mutated patients in FFPE material by a PCR-based assay, with results comparable to those of similar studies. This methodology is reliable, efficient, cost-effective and can be utilised by any standard hospital diagnostic laboratory without the need for any specialized technology such as NGS, RISH or FISH. Given the recent demonstration of intra-tumoral heterogeneity of METex14 status and the lack of suitable antibodies to specifically detect METex14 [8,39], RISH may therefore be suitable for subsequent downstream studies with respect to heterogeneity.

NGS is the current gold standard for identifying METex14, and additionally allows for detection of numerous alterations needed in NSCLC management, and NGS will, in the future, probably be routine for all patients presenting with NSCLC. At present, at least in our institute, the cost for NGS is currently of the order of €400 per patient. Moreover, many hospitals will not have the capacity or resources to conduct NGS, and often, NGS is requested on a case by case basis. We believe that that incorporating a pre-screening step using a simple PCR based assay that can be conducted in most routine hospital laboratories could allow for a cost-effective approach to perhaps pre-screen patients that may respond to MET inhibitors without the need for conducting NGS, or until all patients can have NGS conducted as a routine practice.

Our results suggest that, at least for large scale translational studies, a suitable, cost-effective strategy to screen patients for METEx14 skipping would be via an initial end-point PCR on formalin-fixed paraffin embedded tissue, followed by NGS to confirm diagnosis in those cases testing positive by PCR. Subsequently the issue of intratumoral heterogeneity could be assessed using VAF analysis of NGS data, or RISH could be employed. Finally, our results demonstrate good correlation between all three testing options/modalities (PCR, NGS and RISH).

## Figures and Tables

**Table 1:** Patient samples used in this study.

Patient Specimens	Specimen Type	Modalities Used to Confirm Presence of METex14 Skipping	METex14 Skipping Present
Cohort 1			
Patient 1 (SJH01)	Lung resection—Adenocarcinoma	PCR, RISH, NGS	Y
Patient 2 (SVUH001)	Lung resection—Adenocarcinoma	PCR, RISH, NGS	Y
Patient 3 (SVUH002)	Lung resection—Sarcomatoid carcinoma	PCR, RISH, NGS	Y
Patient 4 (SG01)	Lymph Node—Large cell lung carcinoma	PCR, RISH, NGS	Y
Patient 5 (SG02)	Lung resection—Adenocarcinoma	PCR, RISH, NGS	Y
Patient 6 (SG03)	Lung resection—Undifferentiated-pleomorphic cancer of the lung	PCR, RISH, NGS	Y
Cohort 2			
001	Lung resection—Sarcomatoid carcinoma	PCR	N
002	Lung resection—Sarcomatoid carcinoma	PCR, RISH, NGS	Y
003	Lung resection—Sarcomatoid carcinoma	PCR	N
004	Lung resection—Sarcomatoid carcinoma	PCR	N
005	Lung resection—Sarcomatoid carcinoma	PCR	N
006	Lung resection—Sarcomatoid carcinoma	PCR	N
007	Lung resection—Sarcomatoid carcinoma	PCR	N
008	Lung resection—Sarcomatoid carcinoma	PCR	N
009	Lung resection—Sarcomatoid carcinoma	PCR	N
010	Lung resection—Sarcomatoid carcinoma	PCR	N
011	Lung resection—Sarcomatoid carcinoma	PCR	N
012	Lung resection—Sarcomatoid carcinoma	PCR	N
013	Lung resection—Sarcomatoid carcinoma	PCR	N
014	Lung resection—Sarcomatoid carcinoma	PCR	N
015	Lung resection—Sarcomatoid carcinoma	PCR	N
016	Lung resection—Sarcomatoid carcinoma	PCR	N
017	Lung resection—Sarcomatoid carcinoma	PCR	N
018	Lung resection—Sarcomatoid carcinoma	PCR, RISH, NGS	Y
019	Lung resection—Sarcomatoid carcinoma	PCR	N
020	Lung resection—Sarcomatoid carcinoma	PCR	N

**Table 2:** GBlock dilution for assay sensitivity testing.

Standard	% METex14	METex14 GBlock (10 ng/µL) (µL)	MetWT GBlock (10 ng/µL) (µL)	Total DNA (ng)
1	100	5	0	50
2	75	3.75	1.25	50
3	50	2.5	2.5	50
4	25	1.25	3.75	50
5	10	0.5	4.5	50
6	5	0.25	4.75	50
7	0	0	0	50

**Table 3:** Comparison of common modalities used for the detection of METex14 skipping.

	Immunohistochemistry	PCR	RNA In Situ Hybridisation	Next-Generation Sequencing
Benefits	-Cheap and cost-effective -Rapid result -Easily performed by a standard pathology laboratory -Direct visualisation of tissue	-Rapid result -Reliable, accurate method of detection	-Direct visualisation of tissue -Reliable, accurate method of detection	-High throughput -Reliable, accurate method of detection -Ability to multiplex -Automated analysis
Disadvantages	-Not specific for MET exon 14 skipping splice variant -Heterogeneity of staining has been reported -Interobserver variability	-No direct visualisation of tissue -Easily performed by a standard pathology laboratory	-Expensive reagents -More time consuming if process not automated -Requires multiple sections	-High start-up costs -Dedicated data analysis and storage required -No direct visualisation of tissue -Reduced sensitivity for large insertions or deletions >20 base pairs (bp)
